# Visceral Adipose Tissue Displays Unique Metabolomic Fingerprints in Obesity, Pre-Diabetes and Type 2 Diabetes

**DOI:** 10.3390/ijms22115695

**Published:** 2021-05-27

**Authors:** Tiago Morais, Alexandre L. Seabra, Bárbara G. Patrício, Marta Guimarães, Mário Nora, Pedro F. Oliveira, Marco G. Alves, Mariana P. Monteiro

**Affiliations:** 1 Endocrine and Metabolic Research, Unit for Multidisciplinary Research in Biomedicine (UMIB), University of Porto, 4050-313 Porto, Portugal; tiagoacmorais@gmail.com (T.M.); seabralexandre@hotmail.com (A.L.S.); barbaragomespatricio@gmail.com (B.G.P.); martafilomenaguimaraes@gmail.com (M.G.); mnora1@sapo.pt (M.N.); alvesmarc@gmail.com (M.G.A.); 2 Laboratory for Integrative and Translational Research in Population Health (ITR), University of Porto, 4050-313 Porto, Portugal; 3 Department of Anatomy, Institute of Biomedical Sciences Abel Salazar (ICBAS), University of Porto, 4050-313 Porto, Portugal; 4 Department of General Surgery, Centro Hospitalar de Entre o Douro e Vouga, 4520-220 Santa Maria da Feira, Portugal; 5 QOPNA & LAQV, Department of Chemistry, University of Aveiro, 3810-193 Aveiro, Portugal; pfobox@gmail.com

**Keywords:** obesity, pre-diabetes, type 2 diabetes, adipose tissue, metabolomics

## Abstract

Visceral adipose tissue (VAT) metabolic profiling harbors the potential to disentangle molecular changes underlying obesity-related dysglycemia. In this study, the VAT exometabolome of subjects with obesity and different glycemic statuses are analyzed. The subjects (*n* = 19) are divided into groups according to body mass index and glycemic status: subjects with obesity and euglycemia (Ob+NGT, *n* = 5), subjects with obesity and pre-diabetes (Ob+Pre-T2D, *n* = 5), subjects with obesity and type 2 diabetes under metformin treatment (Ob+T2D, *n* = 5) and subjects without obesity and with euglycemia (Non-Ob, *n* = 4), used as controls. VATs are incubated in culture media and extracellular metabolite content is determined by proton nuclear magnetic resonance (^1^H-NMR). Glucose consumption is not different between the groups. Pyruvate and pyroglutamate consumption are significantly lower in all groups of subjects with obesity compared to Non-Ob, and significantly lower in Ob+Pre-T2D as compared to Ob+NGT. In contrast, isoleucine consumption is significantly higher in all groups of subjects with obesity, particularly in Ob+Pre-T2D, compared to Non-Ob. Acetate production is also significantly lower in Ob+Pre-T2D compared to Non-Ob. In sum, the VAT metabolic fingerprint is associated with pre-diabetes and characterized by higher isoleucine consumption, accompanied by lower acetate production and pyruvate and pyroglutamate consumption. We propose that glucose metabolism follows different fates within the VAT, depending on the individuals’ health status.

## 1. Introduction

Obesity is a major risk factor for pre-diabetes and type 2 diabetes (T2D) [1]. Adipose tissue (AT) maladaptation is believed to lead to obesity-related metabolic disorders such as insulin resistance (IR), pre-diabetes and T2D [2]. These conditions are characterized by a full spectrum of systemic imbalances that culminate in the disruption of glucose homeostasis [3].

From a physiological perspective, processes underlying pre-diabetes and T2D are a continuum that is initiated by resistance to insulin action in mediating glucose transport in peripheral organs, such as the liver, skeletal muscle and AT [4]. As a consequence of decreased glucose uptake, the pancreas is stimulated to increase insulin secretion in an attempt to overcome peripheral resistance [4]. Whenever the pancreatic capacity to sustain insulin hypersecretion is overridden, circulating glucose levels increase and pre-diabetes or overt T2D occurs [4]. In addition, hampered insulin signaling also impairs the suppression of gluconeogenesis and overall mitochondrial function [5,6].

AT presents distinctive functional features depending on regional distribution. In particular, abdominal visceral AT (VAT) is recognized for depicting higher metabolic activity as compared to subcutaneous AT (SAT), as well as having a greater impact on systemic metabolism via the rapid release of free fatty acids [7]. Obesity-related AT dysfunction plays a significant role in the pathogenesis of obesity-related metabolic complications [8,9]. Excessive lipid accumulation within the adipocytes leads to IR, mitochondrial dysfunction and endoplasmic reticulum stress, which are responsible for several unfavorable metabolic adaptations, including decreased oxidative phosphorylation capacity and amino acid (AA) catabolism, along with over activation of gluconeogenesis, a result of the downregulated expression of several genes [10,11]. These metabolic shifts will inevitably influence AT secretome.

Most research has focused on plasma metabolites’ or surrogate markers’ molecular signaling mechanisms, such as AT gene and protein expression [12]. Few studies have addressed AT metabolic signatures ex vivo. System biology comparing AT metabolic profiles across the spectra of increased adiposity, along with dysglycemia, allows us to gain further insights that contribute toward filling the existing knowledge gap. How VAT metabolism impacts glucose homeostasis regulation beyond postprandial glucose uptake in conditions characterized by systemic metabolic dysfunction is not entirely known. Identifying VAT metabolite fingerprints could provide novel insights into the pathogenesis of obesity-related metabolic disorders and potentially disclose molecular targets for intervention.

Thus, the aim of this research work was to characterize the VAT exometabolomic profiles in obesity and co-morbid dysglycemic disorders, namely pre-diabetes and T2D, to gain further insights into putative metabolic shifts and their contributions toward systemic metabolic imbalance.

## 2. Results

### 2.1. Subjects’ Anthropometric and Clinical Features

The study subjects were predominantly female (F:M, 14:5) with a mean age of 49.6 years (range 26–66 years) (Table 1). The body mass index (BMI) in all groups of subjects with obesity was significantly higher compared to Non-Ob (Ob+NGT: 41.4 ± 2.6 kg/m^2^; Ob+Pre-T2D: 44.0 ± 2.8 kg/m^2^; Ob+T2D: 41.5 ± 2.5 kg/m^2^ vs. Non-Ob: 26.1 ± 1.0 kg/m^2^, *p* < 0.001). The fasting glucose levels were significantly higher in Ob+T2D when compared to both Non-Ob and Ob+NGT (Ob+T2D: 161.4 ± 26.8 mg/dL vs. Non-Ob: 88.8 ± 3.2 mg/dL; Ob+NGT: 93.8 ± 0.8 mg/dL, *p* < 0.05). No other significant differences in patient features were found between the study groups at the time of surgery (Table 1).

### 2.2. Metabolite Consumption and Production Rate

#### 2.2.1. VAT Glucose Consumption Was Not Influenced, neither by Obesity nor by Dysglycemia, but Lower VAT Pyruvate Consumption Was Observed in Individuals with Obesity and Pre-Diabetes

**Figure 1:**
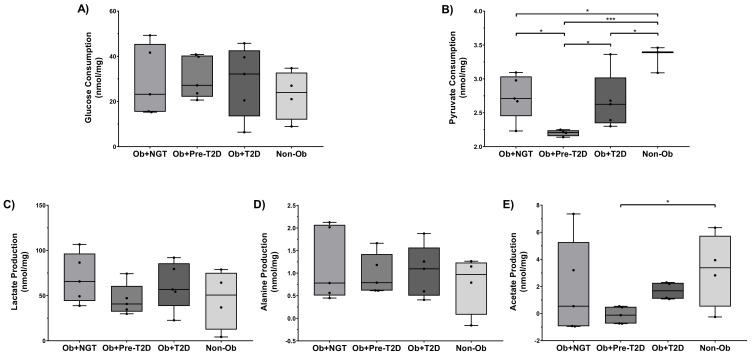
Glucose (**A**) and pyruvate (**B**) consumption and lactate (**C**), acetate (**D**) and alanine (**E**) production by visceral adipose tissue (VAT) explants. Subjects were grouped according to body mass index (BMI) and glycemic status (with obesity and euglycemia-Ob+NGT; with obesity and pre-diabetes-Ob+Pre-T2D; with obesity and T2D-Ob+T2D; without obesity-Non-Ob). Data are presented as boxplots with quartiles and all points plotted. * *p* < 0.05; *** *p* < 0.001.

VAT glucose consumption was not significantly different between the study groups (Figure 1A). However, pyruvate consumption was significantly lower in all groups of subjects with obesity when compared to Non-Ob (Ob+NGT: 2.74 ± 0.14 nmol/mg of wet VAT (WVAT); Ob+Pre-T2D: 2.20 ± 0.02 nmol/mg of WVAT; Ob+T2D: 2.67 ± 0.19 nmol/mg of WVAT vs. Non-Ob: 3.31 ± 0.11 nmol/mg of WVAT, *p* < 0.05). Additionally, pyruvate consumption was also significantly lower in Ob+Pre-T2D when compared to Ob+NGT (Ob+Pre-T2D: 2.20 ± 0.02 nmol/mg of WVAT vs. Ob+NGT: 2.74 ± 0.15 nmol/mg of WVAT, *p* < 0.01) (Figure 1B).

#### 2.2.2. VAT Lactate and Alanine Production Were Not Influenced, neither by Obesity nor by Dysglycemia, but Lower VAT Acetate Production Was Observed in Individuals with Obesity and Pre-Diabetes

No significant differences in lactate and alanine production were identified between the study groups (Figure 1C,D). Acetate production was significantly lower in Ob+Pre-T2D when compared to Non-Ob (Ob+Pre-T2D: −0.12 ± 0.28 nmol/mg of WVAT vs. Non-Ob: 3.21 ± 1.37 nmol/mg of WVAT, *p* < 0.05) (Figure 1E).

#### 2.2.3. A Higher Isoleucine Consumption Was Observed in the VAT of Individuals with Obesity and Pre-Diabetes, Whereas Leucine and Valine Consumption Were Not Influenced by Obesity or Dysglycemia

**Figure 2:**
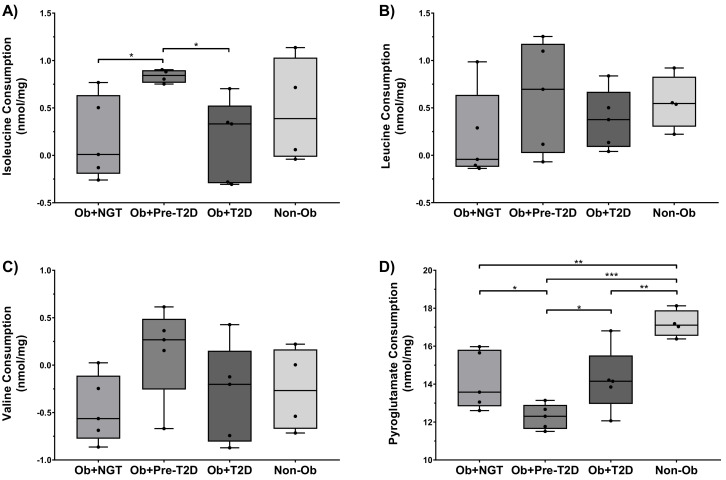
Isoleucine (**A**), leucine (**B**), valine (**C**) and pyroglutamate (**D**) consumption by visceral adipose tissue (VAT) explants. Subjects were grouped according to body mass index (BMI) and glycemic status (with obesity and euglycemia-Ob+NGT; with obesity and pre-diabetes-Ob+Pre-T2D; with obesity and T2D-Ob+T2D; without obesity-Non-Ob). Data are presented as boxplots with quartiles and all points plotted. * *p* < 0.05; ** *p* < 0.01; *** *p* < 0.001.

Isoleucine consumption was significantly higher in Ob+Pre-T2D when compared to Ob+NGT and Ob+T2D (Ob+Pre-T2D: 0.84 ± 0.03 nmol/mg of WVAT vs. Ob+NGT: 0.18 ± 0.20 nmol/mg of WVAT, *p* < 0.05), while no other significant differences in isoleucine consumption were found between the study groups (Figure 2A). Furthermore, no significant differences in leucine or valine consumption were observed between the groups (Figure 2B,C).

#### 2.2.4. Lower Pyroglutamate Consumption Was Observed in the VAT of Individuals with Obesity, Particularly Those with Pre-Diabetes

Pyroglutamate consumption was significantly lower in all groups of subjects with obesity when compared to Non-Ob (Ob+NGT: 14.17 ± 0.69 nmol/mg of WVAT, *p* < 0.01; Ob+Pre-T2D: 12.28 ± 0.30 nmol/mg of WVAT, *p* < 0.001; Ob+T2D: 14.22 ± 0.76 nmol/mg of WVAT, *p* < 0.05 vs. Non-Ob: 17.18 ± 0.36 nmol/mg of WVAT). Furthermore, pyroglutamate consumption was also significantly lower in Ob+Pre-T2D when compared to Ob+NGT (Ob+Pre-T2D: 12.28 ± 0.30 nmol/mg of WVAT vs. Ob+NGT: 14.17 ± 0.69 nmol/mg of WVAT, *p* < 0.01) (Figure 2D).

#### 2.2.5. Pyruvate Consumption Correlates with Several Metabolite Patterns in All Studied Groups, Whereas Glucose Consumption Only Correlates with Alanine Production and Pyroglutamate Consumption in Subjects with Obesity and Pre-Diabetes

In the metabolite profile of Non-Ob, a positive correlation between pyruvate consumption and alanine production was observed (*r* = 0.998) (Table 2).

In contrast, differing metabolite correlation patterns emerged within the metabolite profiles of groups of subjects with obesity. In the metabolite profile of Ob+NGT, in addition to the positive correlation between pyruvate consumption and alanine production (r = 0.958), there was also one with the former and acetate production (r = 0.987), as well as with the former and pyroglutamate consumption (r = 0.916) (Table 2). Several metabolic shifts were observed in Ob+Pre-T2D and Ob+T2D: a significant correlation between pyruvate consumption and alanine production was no longer observed, while a positive correlation between pyruvate consumption and lactate production was observed (Ob+Pre-T2D: r = 0.933; Ob+T2D: r = 0.981) (Table 2). Additionally, a positive correlation between pyruvate consumption and pyroglutamate consumption (r = 0.904) was observed in Ob+Pre-T2D, as well as positive correlations between glucose consumption and alanine production (r = 0.988) and pyroglutamate consumption (r = 0.911). In contrast, in the metabolite profile of Ob+T2D, a negative correlation between pyruvate and isoleucine consumption was observed (r = −0.990) (Table 2).

## 3. Discussion

Tissue metabolomics provide an efficient approach to gathering knowledge on biological statuses through the analysis of intermediary metabolites proximal to the phenotype [13]. In this research, we performed a comprehensive evaluation of AT metabolomics, by qualitatively and quantitatively analyzing the extracellular metabolite shifts of ex-vivo VAT explants from individuals with obesity, obesity plus pre-diabetes and obesity plus T2D.

Our data show that, in the presence of similar insulin concentrations, there are no significant differences in glucose consumption within the VAT across different study groups, further suggesting that impaired glucose uptake is not a prominent VAT feature despite the systemic phenotype, as previously highlighted [14,15]. Nevertheless, in spite of similar glucose consumptions, subjects with obesity, particularly those with pre-diabetes, presented significantly lower VAT pyruvate consumption, along with significantly lower acetate production, suggesting that glucose has different intracellular fates depending on the subjects’ metabolic conditions. Pyruvate is used as a bioenergetics substrate to feed the tricarboxylic acid (TCA) cycle in mitochondria, while acetate has a key role in the molecular processes leading to de novo lipogenesis [16,17,18]. Thus, lower pyruvate consumption could represent an adaptive response to intracellular pyruvate accumulation due to the mitochondrial dysfunction described to be present in omental adipocytes of individuals with obesity [19], while a lower acetate production suggests that there could be increased intracellular consumption, hypothetically derived from a metabolic shift towards de novo lipogenesis. In fact, our new data support our former study findings, in which we showed that obesity is associated with VAT metabolic reprogramming towards de novo lipogenesis [20].

AT AAs’ metabolic signature was previously shown to be modified in the presence of obesity, and even before obesity-related metabolic disorders arise, these changes were noticed to be more profound in VAT [21]. In our current study, considerable changes in VAT AAs’ metabolic signatures were observed across the different study groups. Isoleucine consumption was found to be significantly higher in the VAT of subjects with obesity and pre-diabetes. Isoleucine is an essential branched-chain AA (BCAA) linked to glucose metabolism [22]. Several BCAAs and other essential AAs are known to be involved in glucose homeostasis [23]. AA deamination gives origin to several keto acids that are further metabolized into oxaloacetate and pyruvate, which feed de novo lipogenesis and gluconeogenesis [24], often observed in individuals with pre-diabetes and T2D [24]. In addition, AAs also modulate glucose metabolism in an indirect manner by stimulating insulin and glucagon secretion [24]. Subjects with obesity and compromised metabolic health were described to present impaired BCAA catabolism [8]. Moreover, changes in BCAA catabolism were attributed to modifications in gene expression and protein levels of BCAA-catabolizing enzymes in the VAT [12]. Besides promoting glucose uptake, insulin plays an important role in intracellular metabolism, which includes suppressing gluconeogenesis that becomes hampered in insulin-resistant states, such as pre-diabetes and T2D [10]. Hence, the higher VAT isoleucine consumption observed in individuals with obesity and pre-diabetes could be a compensatory mechanism to retrain the molecular substrates as an indirect sign of inadequate insulin-mediated gluconeogenesis suppression and IR [5,25]. Additionally, BCAAs are catabolized into keto acids by branched-chain aminotransferase (BCAT), which are further oxidized by branched-chain keto acid dehydrogenase (BCKD) into TCA substrates [24]. BCKD defects can derive from enzyme activity disruption in the presence of high fatty acids levels [26]. Due to BCKD’s insufficient activity, accumulated BCAAs are degraded into acylcarnitines that overwhelm beta-oxidation capacity and can further contribute to IR [24,26,27].

Our data also show that pyroglutamate consumption was lower in the presence of obesity, particularly in pre-diabetes. Pyroglutamate is a natural AA derivative of glutamate or glutamine. Glutamate is a vital substrate for energy metabolism that may be used as an alternative energy source to glucose via glycolysis or fatty acids via beta-oxidation [28]. Circulating glutamate levels were demonstrated to be positively associated with several metabolic disorders including IR [6]. High circulating glutamate levels were suggested to derive from reduced cellular uptake, a result of intracellular accumulation of keto-acids, originated from BCAA catabolism [29]. Additionally, high glutamate levels were hypothesized to contribute to disease progression since glutamate decreases insulin secretion [24] and stimulates glucagon release from pancreatic alpha cells, which in turn promotes pyruvate to alanine transamination and gluconeogenesis [30]. Indeed, alanine and glutamine are among the most important gluconeogenic precursors [31,32]. Furthermore, considering that the first step of BCAA catabolism produces glutamate, decreased pyroglutamate consumption in the VAT of individuals with obesity and pre-diabetes could also represent an adaptive phenomenon to the intracellular accumulation of glutamate, leading to the downregulation of glutamate uptake, thus linking isoleucine to glutamate [8].

From a systemic perspective, the profound differences found in VAT AA metabolism, with blunted AA metabolism and overload of BCAA catabolism, may also contribute to determining the circulatory flux of AA with gluconeogenic potential to other peripheral organs involved in glucose metabolism [8,9,29,33].

In a large meta-analysis, the circulating AA profiles of individuals with obesity, metabolic syndrome and T2D were demonstrated to differ from normal-weight healthy controls in different magnitudes, with the greatest differences being observed in patients with obesity followed by those with metabolic syndrome and T2D. In subjects presenting these metabolic disorders, circulating AA profiles were characterized by increased valine, isoleucine, glutamate and proline levels, while glycine levels were decreased [29]. Moreover, plasma BCAA levels were found to be positively correlated with VAT and systemic markers of IR, which suggests that AT could be involved in BCAA homeostasis and IR [9]. More recently, BCAAs, isoleucine, valine and leucine were also demonstrated to be associated with decreased insulin secretion [33], while several other metabolites including BCAAs and aromatic AAs were shown to be associated with T2D risk [34]. Overall, the available evidence suggests that abnormal BCAA metabolism may be a downstream mediator of adiposity and IR that precedes hyperglycemia on the causal pathway to T2D [35].

In contrast, our study shows that the VAT metabolomic profile of subjects with obesity and T2D has more resemblance to that of individuals with obesity and euglycemia than that of those with pre-diabetes. Of notice, all subjects with T2D were under treatment with the glucose-lowering drug metformin until the day of VAT sampling during the elective surgical procedure. Metformin is an insulin-sensitizing drug used as a first-line T2D treatment [36]. Several insulin-sensitizing interventions, including weight-loss surgery and physical exercise, were demonstrated to be able to modify metabolic profiles, including BCAA and glutamate levels [37,38]. In addition, metformin is recognized for having beneficial effects of VAT reduction, which were previously attributed to a potential shift in fuel resource into fat oxidation [39] and an upregulation of adaptive thermogenesis [40]. In fact, metformin was shown to alter adipocyte metabolism by reducing cellular oxygen consumption in a dose-dependent manner in cultured human brown adipocyte cells [41]. Metformin decreases gluconeogenesis by inhibiting glycerophosphate dehydrogenase and activating AMPK, which suppresses mTORC1 on the mTOR pathway, responsible for triggering a selective pattern of IR within the AT with hampered insulin-mediated gluconeogenesis suppression, yet preserved lipogenesis [39,42]. Thus, our findings suggest that metformin could be responsible for the reversal of the VAT metabolic profile of individuals with T2D toward a profile that resembles the one observed in subjects with obesity but without altered glucose metabolism—concerning the pyruvate and isoleucine profiles—suggesting improved gluconeogenesis suppression.

Inevitably, this study presents some limitations that must be acknowledged. One of our study limitations is the fact that our control group should be comprised of normal-weight and metabolically healthy subjects, yet our controls do not meet the ideal criteria. This is mainly attributed to the fact that VAT can only be collected during an elective laparotomy or laparoscopic surgery, which healthy subjects without acute infectious/inflammatory or active neoplastic conditions are unlikely to require. Therefore, patients with biliary calculus undergoing prophylactic cholecystectomy were selected for study enrolment as controls. Given that this patient population often presents excess weight and dyslipidemia as comorbid conditions, the “non-obesity” group is unsurprisingly biased, despite presenting clear phenotypic differences when compared to the other groups. Another limitation of our study is the fact that our patients did not undergo an extensive assessment of their glycemic status, as only routine pre-operative biochemical evaluations according to standard practice were conducted, which included fasting glucose, and HbA1c level measurement whenever fasting glucose was found to be elevated. Given the fact that patients did not undergo oral glucose tolerance tests to further characterize their glycemic status, we are aware that an HbA1c under 6.5% does not completely exclude the diagnosis of overt T2D, although renders it is less likely as compared to individuals with elevated HbA1c. Nevertheless, we believe that these limitations do not invalidate our finding when comparing between groups, since subjects presented clear phenotypic differences to allow the comparisons that were made. Additionally, our data rely on the analysis of extracellular metabolic profiles, which can only provide a limited perception of intracellular metabolomic dynamics. Furthermore, the current study did not include lipidomic analysis that could provide additional insights into the adipocyte metabolic profile, as lipogenesis and lipolysis are the core VAT functions. Our interpretation of the metabolic pathway modifications within the VAT of individuals with obesity and dysglycemia can only be considered hypothesis-generating, to be explored in future studies—namely, those focusing on intracellular intermediate metabolism, mitochondrial function and metformin actions—to pinpoint the molecular disruptions associated with these disease states. Finally, we used a metabolomic analysis, which has lower sensitivity when compared to mass spectrometry, which limited the span of metabolites that could be analyzed.

Nonetheless, the data retrieved from this research build on the previous knowledge of VAT metabolic dynamics since the majority of the studies previously reported were either mostly focused on characterizing plasma metabolites, as surrogate markers of adiposity dysfunction [43]; were focused on AT metabolomics or mitochondrial function pertaining to subjects with obesity but not compared to the full spectrum of subjects with obesity comorbidities, such as pre-diabetes and T2D, nor under the influence of metformin [21]; or instead of intact VAT explants, used in vitro differentiated cells derived from isolated pre-adipocytes for the metabolomics analysis, which are subjected to interferences that could alter cell metabolic behavior [44]. In contrast, the herein described study was conducted in VAT explants that represent a closer resemblance to in vivo function with no selective loss of any cell subtype, while using a methodology (1H-NMR) that allows the assessment of small metabolites, such as acetate and pyruvate, which would be missed by other techniques such as gas chromatography/mass spectrometry [45]. For the first time, to the best of our knowledge, our study has highlighted the extracellular metabolite shifts of ex-vivo VAT explants from individuals with obesity, obesity plus pre-diabetes and obesity plus T2D, demonstrating that there is continuous VAT metabolic dysfunction along the spectrum of conditions spanning from excess weight towards obesity-associated dysglycemia. Moreover, the metabolomic signatures suggest that the VAT of individuals with obesity-associated pre-diabetes is characterized by increased gluconeogenic drive and decreased mitochondrial oxidative capacity, which seem to be imprinted and are retained ex-vivo despite being in the presence of similar extracellular stimuli. This novel finding further supports previous findings that described the downregulation of mitochondrial protein complexes, mitochondrial DNA and oxidative capacity in the AT of subjects with acquired obesity, even prior to the onset of metabolic complications [46,47,48].

**Figure 3:**
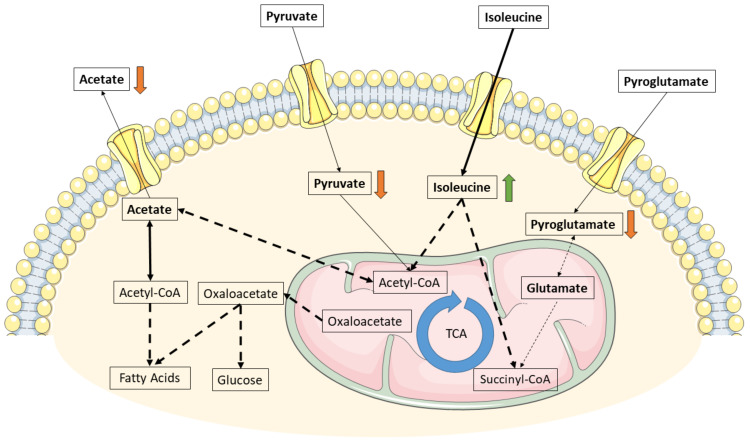
Putative metabolic fates of acetate, pyruvate, isoleucine and pyroglutamate in the VAT of subjects with obesity and pre-diabetes. Isoleucine consumption feeds the increased gluconeogenic flux and the tricarboxylic-acid cycle (TCA), compensating for the lower influx of pyruvate and pyroglutamate. Lower acetate production to the extracellular medium could derive from increased intracellular consumption to promote de novo lipogenesis. Thicker arrows show increased flux; dashed lines denote simplified pathways.

In sum, our study findings enable us to define a metabolic fingerprint of VAT associated with pre-diabetes, with a higher isoleucine consumption accompanied by a lower acetate production and pyruvate and pyroglutamate consumption (Figure 3). These results provide an overview of intermediate metabolism in the VAT, which contributes toward the systemic phenotype observed in these individuals. Moreover, our data suggest that glucose follows different fates within the VAT depending on the individuals’ metabolic health.

## 4. Materials and Methods

### 4.1. Subjects

Subjects planning to undergo elective laparoscopic surgical procedures for the primary treatment of hiatal esophageal hernia, gallbladder stones or bariatric surgery at a single public academic hospital were invited to take part in the study. Those patients who accepted to participate and did not have any of the pre-established exclusion criteria—namely, ongoing pregnancy, active acute infectious conditions or prior history of neoplastic diseases—were included.

Study subjects (N = 19) were allocated into groups according to BMI, into a group of subjects with obesity (*n* = 15) and another group of subjects without obesity and with euglycemia, used as controls (Non-Ob, *n* = 4). Subjects with obesity were further stratified into three subgroups according to HbA1c levels: subjects with HbA1c under 5.6% were considered as having euglycemia (Ob+NGT, *n* = 5), subjects with HbA1c between 5.6% and 6.4% were considered as having pre-diabetes (Ob+Pre-T2D, *n* = 5) and subjects with HbA1c over 6.4% were considered as having T2D (Ob+T2D, *n* = 5). All subjects enrolled in the study with a T2D diagnosis were under metformin treatment until the day before surgery.

All study procedures were approved by the Intuitional Ethics Committee (CA 0830/16-Ot, CHEDV-HSS) and conducted in accordance with the Local, National and European Ethical Guidelines for Medical Research involving Human Subjects. All patients signed the informed consent form before surgery and AT sampling.

### 4.2. Adipose Tissue Isolation and Explants Incubation

VAT harvested after a minimum 12 h fast, under sterile conditions during laparoscopic surgical procedures, was immediately processed to remove macroscopically visible damaged tissue, weighed and divided into fragments with approximate weights of 20 mg.

VAT fragments were placed into 48-well plates and left to acclimatize in 200 µL DMEM/F12 (12-719F, Lonza, Basel, Switzerland) in a cell culture chamber at 37 °C in the presence of 5% CO_2_ for 1 h. After the acclimatization period, VAT culture media were replaced by fresh culture media and allowed to rest for an additional period of 1 h. Afterward, VAT culture media were again replaced by fresh culture media supplemented with insulin (100 nM, Actrapid, Novo Nordisk, Bagsværd, Denmark) and 1% penicillin-streptomycin (P4333, Sigma-Aldrich, St. Louis, MO, USA). The VAT explants’ culture media were collected 48 h later and stored at −20 °C for later analysis.

### 4.3. Proton Nuclear Magnetic Resonance (^1^H-NMR)

The metabolite contents of the collected VAT culture media were determined by ^1^H-NMR, as previously described [20]. As an internal standard, 1 mM of sodium fumarate (Sigma-Aldrich, St. Louis, MO, USA) was used (singlet, 6.50 ppm).

Spectra analysis enabled us to identify and quantify the following metabolites (multiplicity, chemical shift): H1-α-glucose (doublet, 5.22 ppm), pyroglutamate (doublet of doublets, 4.16 ppm), pyruvate (singlet, 2.38 ppm), acetate (singlet, 1.90 ppm), alanine (doublet, 1.44 ppm), lactate (doublet, 1.33 ppm), isoleucine (doublet, 0.99 ppm) and valine (doublet, 0.97 ppm). The relative areas of ^1^H-NMR resonances were quantified using peak area integration with NUTS-Pro (Acorn NMR, Livermore, CA, USA). The results are expressed as nanomoles of metabolite consumed/produced per milligram of WVAT.

### 4.4. Statistical Analysis

All data are presented as mean ± standard error of the mean (SEM). Outliers were identified using the ROUT method (Q = 5%). The Shapiro-Wilk normality test was used to determine the normality of the groups. Comparison of independent groups was carried out using an ordinary one-way ANOVA test paired with Fisher’s LSD test. The association between metabolite consumption/production and BMI was evaluated by computing Pearson correlation coefficients or Spearman’s rank correlation coefficients, depending on the normality of the data, at a confidence interval of 95%. All *p* values < 0.05 were considered statistically significant. Statistical analysis was performed using GraphPad Prism 8.0.1 (GraphPad Software Inc., San Diego, CA, USA) and SPSS 27 (IBM Corporation, Armonk, NY, USA).

## Figures and Tables

**Table 1:** Study subjects’ anthropometric, clinical and biochemical features.

	Ob+NGT (*n* = 5)	Ob+Pre-T2D (*n* = 5)	Ob+T2D (*n* = 5)	Non-Ob (*n* = 4)
Age (years)	44 ± 7	50 ± 3	56 ± 2	48 ± 7
Sex (F:M)	4:1	4:1	4:1	2:2
BMI (kg/m^2^)	41.4 ± 2.6 ***	44.0 ± 2.8 ***	41.5 ± 2.5 ***	26.1 ± 1.0
Fasting glucose (mg/dL)	93.8 ± 0.8	109.2 ± 8.0	161.4 ± 26.8 *^,†^	88.8 ± 3.2
HbA1c (%)	5.4 ± 0.2	6.2 ± 0.2	7.6 ± 1.3 ^†^	Not available
Metformin (%)	0%	0%	100%	0%
SBP (mmHg)	137 ± 7	150 ± 6	146 ± 4	133 ± 7
DBP (mmHg)	79 ± 4	85 ± 3	73 ± 4	80 ± 4
Total cholesterol (mg/dL)	161 ± 7	220 ± 15	206 ± 24	177 ± 25
HDL (mg/dL)	52 ± 5	47 ± 7	48 ± 5	35 ± 11
LDL (mg/dL)	96.4 ± 10.3	142.4 ± 7.2	135.4 ± 23.2	100.9 ± 6.7
Triglycerides (mg/dL)	102 ± 8	111 ± 22	203 ± 49	206 ± 147

Subjects were grouped according to body mass index (BMI) and glycemic status (with obesity and euglycemia—Ob+NGT; with obesity and pre-diabetes—Ob+Pre-T2D; with obesity and T2D—Ob+T2D; without obesity—Non-Ob). HbA1c—hemoglobin A1c; SBP—systolic blood pressure; DBP—diastolic blood pressure; HDL—high-density lipoprotein; LDL—low-density lipoprotein. Data are presented as mean ± SEM. * vs. Non-Ob (*, *p* < 0.05; ***, *p* < 0.001); ^†^ vs. Ob+NGT (^†^, *p* < 0.05).

**Table 2:** Pearson correlation coefficients between glucose and pyruvate consumption and metabolite flux.

	Non-Ob (*n* = 4)						
	Lactate (P)	Acetate (P)	Alanine (P)	Isoleucine (C)	Leucine (C)	Valine (C)	Pyroglutamate (C)
Glucose (C)	0.839	0.457	0.788	0.742	0.660	0.681	0.511
Pyruvate (C)	0.869	0.367	0.998 *	−0.887	−0.991	−0.794	−0.023
	Ob+NGT (*n* = 5)						
Glucose (C)	0.844	0.330	0.424	0.264	−0.061	0.316	0.553
Pyruvate (C)	0.617	0.987 *	0.958 *	−0.751	−0.729	−0.742	0.916 *
	Ob+Pre−T2D (*n* = 5)						
Glucose (C)	0.860	−0.909	0.988 **	0.660	0.868	0.394	0.911 *
Pyruvate (C)	0.933 *	−0.469	0.734	0.064	0.825	−0.354	0.904 *
	Ob+T2D (*n* = 5)						
Glucose (C)	−0.009	−0.805	0.638	0.177	0.573	0.357	0.152
Pyruvate (C)	0.981 **	0.470	0.281	−0.990 **	−0.527	−0.936 *	0.761

(C)—metabolite consumption; (P)—metabolite production. Subjects were grouped according to body mass index (BMI) and glycemic status (with obesity and euglycemia—Ob+NGT; with obesity and pre-diabetes—Ob+Pre-T2D; with obesity and T2D—Ob+T2D; without obesity—Non-Ob). Spearman’s *p* value (*, *p* < 0.05; **, *p* < 0.01).

## Data Availability

The data that support the findings of this study are available from the corresponding author upon reasonable request.
